# Psychological Stress-Induced, IDO1-Dependent Tryptophan Catabolism: Implications on Immunosuppression in Mice and Humans

**DOI:** 10.1371/journal.pone.0011825

**Published:** 2010-07-28

**Authors:** Cornelia Kiank, Jan-Philip Zeden, Solveig Drude, Grazyna Domanska, Gerhard Fusch, Winfried Otten, Christine Schuett

**Affiliations:** 1 Department of Immunology, DFG Graduate School GK840, Ernst Moritz Arndt University, Greifswald, Germany; 2 Department of Pediatrics, Ernst-Moritz-Arndt University of Greifswald, Greifswald, Germany; 3 Research Unit Behavioural Physiology, Research Institute for the Biology of Farm Animals (FBN), Dummerstorf, Germany; New York University, United States of America

## Abstract

It is increasingly recognized that psychological stress influences inflammatory responses and mood. Here, we investigated whether psychological stress (combined acoustic and restraint stress) activates the tryptophan (Trp) catabolizing enzyme indoleamine 2,3-dioxygenase 1(IDO1) and thereby alters the immune homeostasis and behavior in mice. We measured IDO1 mRNA expression and plasma levels of Trp catabolites after a single 2-h stress session and in repeatedly stressed (4.5-days stress, 2-h twice a day) naïve BALB/c mice. A role of cytokines in acute stress-induced IDO1 activation was studied after IFNγ and TNFα blockade and in IDO1^−/−^ mice. RU486 and 1-Methyl-L-tryptophan (1-MT) were used to study role of glucocorticoids and IDO1 on Trp depletion in altering the immune and behavioral response in repeatedly stressed animals. Clinical relevance was addressed by analyzing IDO1 activity in patients expecting abdominal surgery. Acute stress increased the IDO1 mRNA expression in brain, lung, spleen and Peyer's patches (max. 14.1±4.9-fold in brain 6-h after stress) and resulted in a transient depletion of Trp (−25.2±6.6%) and serotonin (−27.3±4.6%) from the plasma measured 6-h after stress while kynurenine levels increased 6-h later (11.2±9.3%). IDO1 mRNA up-regulation was blocked by anti-TNFα and anti-IFNγ treatment. Continuous IDO1 blockade by 1-MT but not RU486 treatment normalized the anti-bacterial defense and attenuated increased IL-10 inducibility in splenocytes after repeated stress as it reduced the loss of body weight and behavioral alterations. Moreover, kynurenic acid which remained increased in 1-MT treated repeatedly stressed mice was identified to reduce the TNFα inducibility of splenocytes *in vitro* and i*n vivo*. Thus, psychological stress stimulates cytokine-driven IDO1 activation and Trp depletion which seems to have a central role for developing stress-induced immunosuppression and behavioral alteration. Since patients showed Trp catabolism already prior to surgery, IDO is also a possible target enzyme for humans modulating immune homeostasis and mood.

## Introduction

There is increasing understanding that social and psychological stressors such as anxiety, social isolation or insecurity affect health. Stressed individuals become more susceptible to infection, tumors, hypertension, heart attack, stroke, autoimmunity or affective disorders. Depression, for example, was the 4^th^ leading contributor to the global burden of disease in 2000 and was projected to be in second place in 2020 [1]–[5].

Physical, chemical and emotional stress stimulate the autonomous nervous system including the efferent sympathetic and vagal pathways and the hypothalamic-pituitary-adrenal (HPA) axis resulting in glucocorticoid (GC) secretion into the circulation [3]. A prolonged stimulation of the stress axes causes long-term neuroendocrine disturbances which are well characterized [6]–[10] but vary depending on the individual's genetics and environmental factors, affecting fetal and postnatal neurochemical and endocrine differentiation [11].

Adapted from a murine model where mechanisms of stress-induced reactivation of colitis was studied [12], we recently showed that repeated combined acoustic and restraint stress, which is a model of severe stress involving physical restraint and emotional stress, induces depression-like alterations and immunosuppression in BALB/c mice. Stress-induced alterations included an increased lymphocyte apoptosis, an anti-inflammatory cytokine bias and a reduced anti-bacterial response [8], [13]. Spontaneous dissemination of bacteria into extra-intestinal sites which is found in repeatedly stressed mice [13] may be of gut origin since stress sensitive intestinal “leakage” was described for both, the large and small intestine [14]–[19].

**Figure 1:**
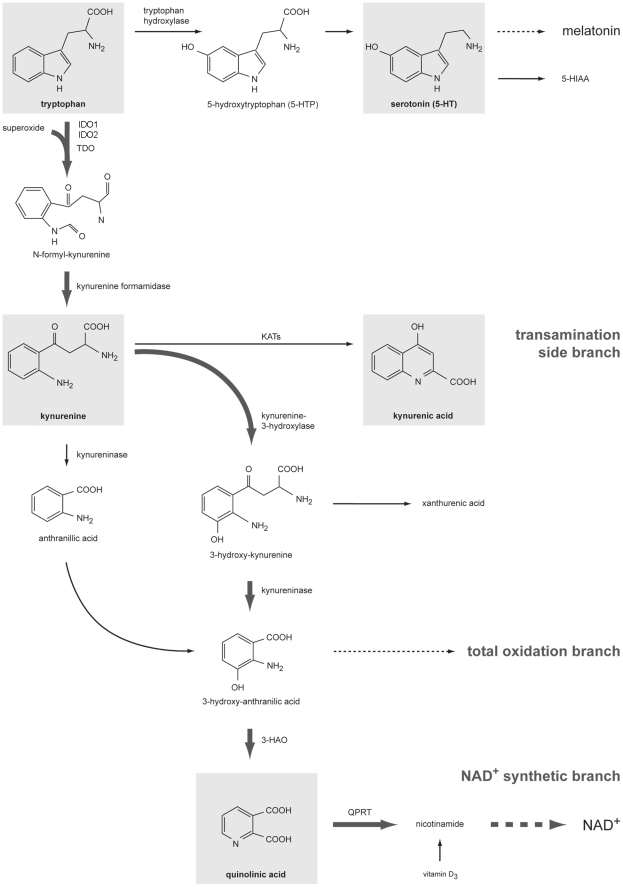
Major pathways of tryptophan (Trp) catabolism. Dietary Trp is essential for protein biosynthesis, but the majority is catabolized along the kynurenine pathway (bold arrows) providing NAD^+^ for cellular redox- and energy homeostasis. Alternative pathways are the conversion of Trp to serotonin (serotonin) and then to melatonin, or to tryptamine and then to the kyurenamines (not shown). Indoleamines analysed in this study are shaded in grey. Abbreviations: 3-HAO, 3-hydrox-anthranilic acid oxidase; 5-HIAA, 5-hydroxy-indole acetic acid; IDO1 and 2, indoleamine 2,3-dioxgenase 1 and 2; KATs, kynurenine aminotransferases; NAD+,nicotinamide adenine dinucleotide; MAO monoamine oxidase; QPRT, quinolinic acid phosphoribosyltransferase; TDO, tryptophan 2,3-dioxygenase.

The consequences of such bacterial translocation on immune function and behavior are not entirely characterized. Interestingly, both immune and behavioral regulatory systems [20]–[24] as well as bacterial growth [25], [26] depend on the availability of tryptophan (Trp). Under basal conditions in mammals, Trp catabolism is mediated by tryptophan dioxygenase (TDO) activation - mainly in the liver - which is controlled by GCs [22], [27]. Additional Trp catabolic pathways can be recruited by the activation of indoleamine 2,3-dioxygenase 1 (IDO1). IDO1 is inducible by bacterial products (LPS, CpG) and proinflammatory mediators including TNFα or IFNγ [22], [27], [28] and is susceptible for various internal and external stimuli, such as hormones, stress and immune activation [29]. It was demonstrated that increased tryptophan depletion via the IDO1 pathway increases the generation of kynurenines (see Fig. 1) which inhibit T cell responses and cause the development of dendritic cells with tolerogenic properties [22], [27].

In addition, mammals use 1% of dietary tryptophan for the synthesis of serotonin (5-HT, see Fig. 1). About 10% of this conversion is found in the brain [29]. When Trp as the precursor of serotonin is depleted, mood alterations develop even in healthy humans [30], [31]. Moreover, there is increasing evidence that the activation of the IDO1-driven Trp catabolic pathway has clinical implication for humans: shown for sepsis or trauma [32], [33], depression [34], [35] and schizophrenia [34].

Bringing together that bacterial products potentially induce IDO1 activity [20], [22], [28], [36] and that repeatedly stressed BALB/c mice show an increased bacterial burden, an anti-inflammatory cytokine bias and behavioral alteration as well as Trp shortage [7], [8], [13], we hypothesize that stress induces IDO1 activity.

Therefore, the aims of this study were to identify whether stress exposure results in IDO1-dependent Trp catabolism and whether this mediates immunosuppression and behavioral alterations in repeatedly stressed mice. Finally, we conducted a pilot study to analyze whether similar pathways are activated in humans undergoing surgery.

## Results

### Enhanced tryptophan catabolism in repeatedly stressed mice

The analysis of Trp-kynurenine pathway metabolites in repeatedly stressed mice showed that plasma Trp and serotonin levels were reduced and Kyna accumulated while other kynurenines were not altered (Table 1). Preliminary data give evidence that ip injection of Kyn is followed by an increase of plasma Kyna levels within the next 30 minutes whereas peripheral Quin levels remain unaffected in BALB/c mice (Suppl. Fig. S1), indicating that Kyna accumulates in mice whereas Quin may be rapidly catabolized for NAD^+^ synthesis (see Fig. S1).

**Table 1:** Tryptophan and serotonin shortage, as well as increased concentrations of kynurenic acid in the plasma of repeatedly stressed mice compared with non-stressed controls.

metabolite [µmol/l]	repeatedly stressed	non-stressed	p
tryptophan (Trp)	40.03±3.98	49.15±5.03	.0003
kynurenine (Kyn)	1.68±0.34	1.80±0.65	n.s.
kynurenic acid (Kyna)	0.90±0.39	0.53±0.10	.024
quinolinic acid (Quin)	0.25±0.03	0.25±0.04	n.s.
serotonin (5-HT)	1.82±0.32	2.2±0.31	.0003

n = 9 mice/group, comparison of repeatedly stressed and non-stressed female BALB/c mice by Mann-Whitney U-test, n.s. = not significant

We blocked GC-receptors using RU486, to study the role of glucorticoids (GCs) on Trp catabolism and immunosuppression in stressed mice. RU486 treatment did not prevent Trp depletion (Suppl. Fig. S2A) and failed to restore the antibacterial defense in repeatedly stressed animals (Suppl. Fig. S2B,C) – even resulted in a higher bacterial load in the blood 24-h after experimental infection with *E. coli* (Suppl. Fig. S1C) which indicates that other mediators than GCs mediate activation of the Trp-kynurenine catabolic pathway in BALB/c mice.

### Stress-induced IDO1 activation depends on IFNγ and TNFα

Knowing that acute stress alters the intestinal barrier function and induce a systemic low-grade inflammation in BALB/c mice [37], we thought that this may active IDO1-dependent Trp catabolism in these animals.

**Figure 2:**
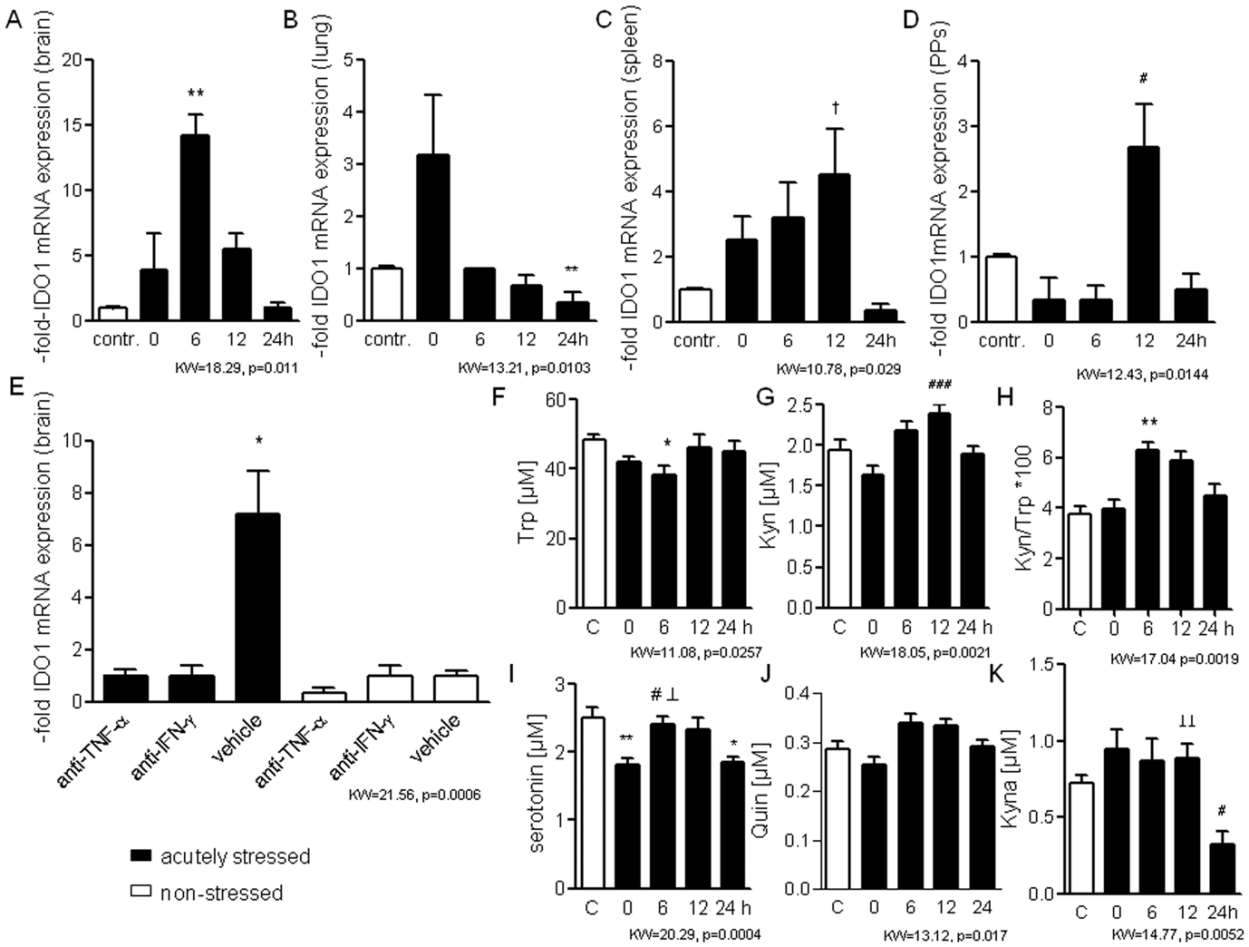
Transient activation of Trp catabolism after acute psychological stress. **A–E.** Kinetics of IDO1 mRNA expression induced in brain (A), lung (B), spleen (C) and Peyer's patches (D) within 24-h after termination of 2-h-stress exposure (n = 6 mice/time, n = 6 controls; average values of basal expression levels in non-stressed mice were assigned as value of 1), and IDO1 mRNA expression in the brain of TNFα antiserum- and IFNγ antiserum treated vs. vehicle-treated animals 6-h after acute stress (E, n = 6 mice/group, average values of basal expression levels in vehicle treated, non-stressed mice assigned as value of 1). **F–I.** Kinetics of plasma concentrations of Trp (F), Kyn (G), the Kyn/Trp ratio (H) and of the levels of serotonin (I), Quin (J) and Kyna (K) following 2-h-stress exposure (n = 6 mice/time) compared with basal levels of healthy controls (n = 15 mice/group). All panels depict data of one representative experiment of two: *p<.05; **p<.01; ***p<.001 compared with non-stressed controls; ^#^p<.05; ^##^p<.01; ^###^p<.001 compared with mice immediately after acute stress (0-h) and ^⊥^<.05 ^⊥⊥^<0.01, ^⊥⊥⊥^<0.001 compared with mice 24-h after stress exposure by Kruskal-Wallis testing with *post-hoc* Dunn Multiple comparison testing (KW- and p-values are indicated in the graph).

Indeed, we found transiently increased IDO1 mRNA expression in several tissues (whole organ homogenates) with a highest response in the brain (14.17±3.83-fold) 6-h after acute stress (Fig. 2A). An up-regulation of IDO1 mRNA expression was also measured in the lung (3.17±2.79-fold) immediately after acute stress (Fig. 2B), as well as in the spleen (4.33±3.67-fold) and in isolated Peyer's patches (2.67±1.63-fold) 12-h after termination of stress (Fig. 2C,D). One day later, IDO1 mRNA levels were back to normal (Fig. 2A–D). The detailed mechanisms for the different time kinetics of IDO1 induction in different organs remains to be elucidated.

Both TNFα and IFNγ antibody treatments blocked the induction of IDO1 mRNA in the brain (Fig. 2E) indicating that a proinflammatory response to stress is essential for IDO1 induction.

Importantly, we showed that IDO1 is not only expressed on the mRNA level but is enzymatically active leading to an early decrease of plasma Trp (Fig. 2F) and serotonin levels (Fig. 2I) and transiently increased Kyn concentrations at 6- and 12-h after acute stress (Fig. 2G) whereas Quin levels were not significantly changed (Fig. 2J) and Kyna levels were not significantly altered until 12-h after stress exposure (Fig. 2K). The Kyn/Trp ratio indicating IDO1-driven metabolism was significantly increased at 6-h after termination of stress (Fig. 2H). One day after acute stress, plasma Trp and Kyn were back to normal whereas Kyna and serotonin concentrations were significantly decreased (Fig. 2F–K).

### Blocking IDO1 activity abrogates chronic stress-induced immunosuppression

Since GCs did not mediate repeated stress-induced loss of the anti-bacterial defense and we found that already acute stress induces IDO1 expression, we next studied whether the activation of the IDO1-dependent Trp catabolic pathway affects repeated stress-induced immunosuppression and depression-like behavior by competitively inhibiting IDO1 with 1-MT. After 5 days 1-MT treatment, we measured pharmacological concentrations of 33.43±7.18 µmol/l 1-MT in the plasma (n = 6 healthy female BALB/c mice) which was associated with an increased serotonin turnover in the brain of healthy mice (Suppl. Fig. S3).

**Figure 3:**
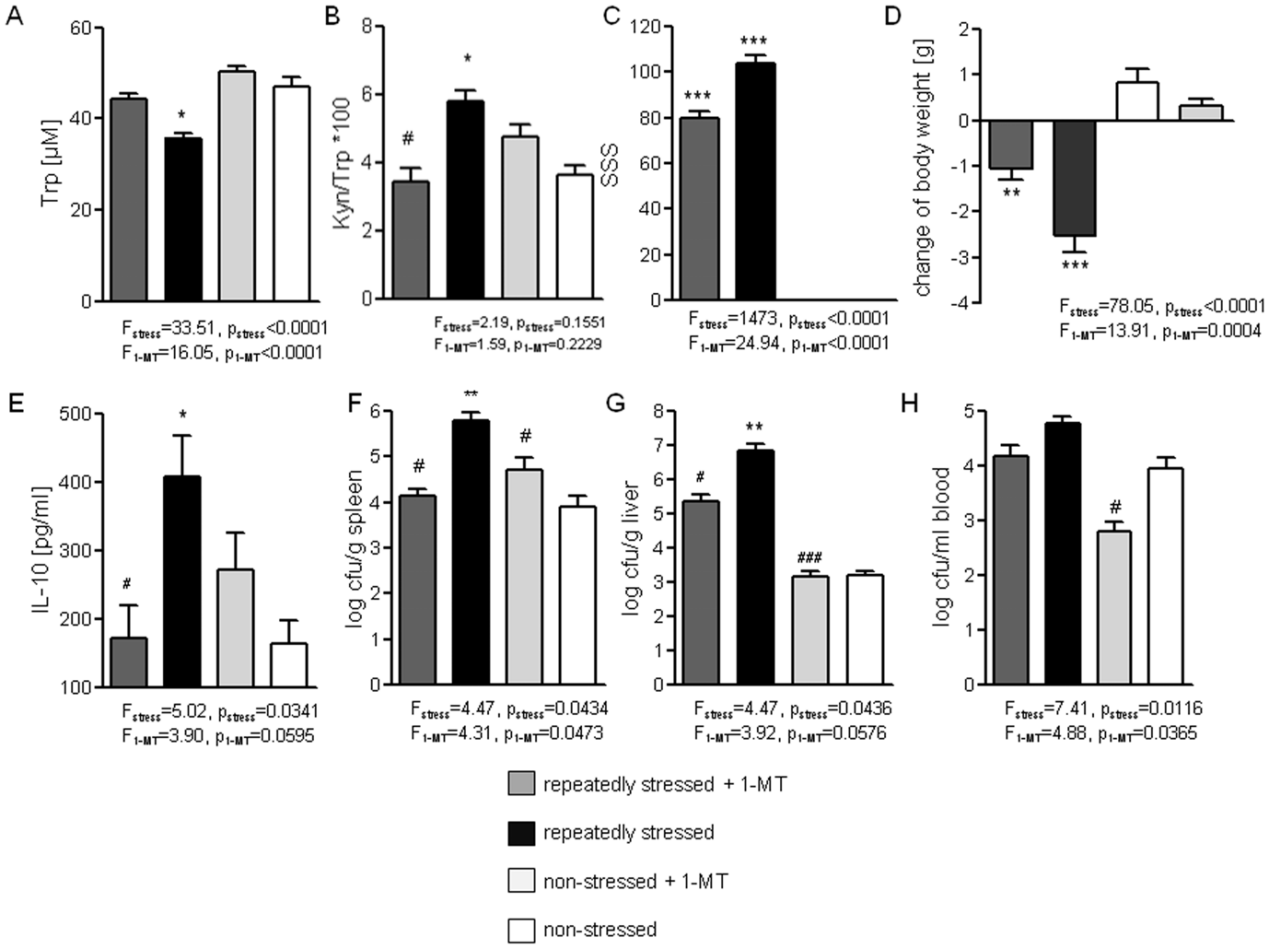
Effects of pharmacological IDO1 blockade on Trp catabolism, IL-10 inducibility and anti-bacterial defense in repeatedly stressed mice. **A–E.** Plasma concentrations of tryptophan (A) and the Trp/Kyn ratio (B), Stress Severity Score evaluating the behavioral stress response (C), change of body weight during repeated stress (D) and IL-10 levels in the supernatant of splenocytes after *ex vivo* stimulation with LPS (*from S. abortus equi*, 1-µg/ml) (E) of repeatedly stressed and non-stressed mice, with or without 1-MT treatment which were measured immediately after the termination after the ninth stress exposure. **F–H.** Bacterial burden in spleen (F), liver (G) and blood (H) 24-h after ip infection with *E. coli* ATCC 25922 in mice with or without 1-MT treatment after repeated stress and of non-stressed mice (injection was performed immediately after the ninth stress exposure); n = 8 mice/group, summary of two independent experiments; *p<.05, **p<.01, ***p<.001 compared with non-stressed, without 1-MT-treatment, ^#^p<.05; ^##^p<.01; ^###^p<.001 compared with repeatedly stressed mice by Kruskal-Wallis testing with *post-hoc* Dunn's Multiple comparison testing. Two-way ANOVA and *post-hoc* Bonferroni's Multiple comparison test were used for testing the overall influences of stress and treatment (ANOVA F- and p-values for the influence of stress and 1-MT are indicated in the graph).

IDO1 blockade reduced the drop-down of plasma Trp levels in repeatedly stressed mice which normalized the Kyn/Trp ratio (Figure 3A,B). We found that repeatedly stressed mice without treatment showed significantly reduced serotonin levels (Tab. 1) whereas 1-MT treated, stressed mice showed no difference compared with non-stressed control animals (data not shown) and by trend higher levels than the non-treated, stressed group (2.0±0.05 µM in 1-MT treated vs. 1.79±0.07 µM in non-treated repeatedly stressed mice; n = 12 mice, p = 0.06).

IDO blockade attenuated the behavioral stress response which is reflected by a reduced Stress Severity Score (SSS, Fig. 3C). The SSS assesses the individual reaction of mice using a standardized protocol including the evaluated of urination, defecation/diarrhea, fur condition/grooming, reduction of activity/huddling behavior and reduction of muscle tone. We found that 1-MT treatment primarily increased the activity of mice and prevented the loss of exploratory behavior after repeated stress (data not shown). 1-MT treated mice also did not lose as much body weight as repeatedly stressed mice without 1-MT did (Fig. 3D).

*In vivo* 1-MT treatment attenuated the increased IL-10 inducibility in repeatedly stressed mice (Fig. 3E), which was measured in the supernatant of splenocyte cultures after 24-h of *ex vivo* stimulation with LPS. Most importantly, IDO blockade improved the anti-bacterial defense which is shown by a reduced stress-induced increase of the bacterial load of spleen, liver and blood 24-h after experimental infection with *E. coli* (Fig. 3F–H). Supportively, IDO1-deficient repeatedly stressed mice display a by trend reduced bacterial dissemination into the liver (Suppl. Fig. 4A) and a significantly reduced bacteremia 24-h after experimental infection with *E. coli* (Suppl. Fig. S4B).

### Kynurenic acid blocks TNFα inducibility in splenocytes

**Figure 4:**
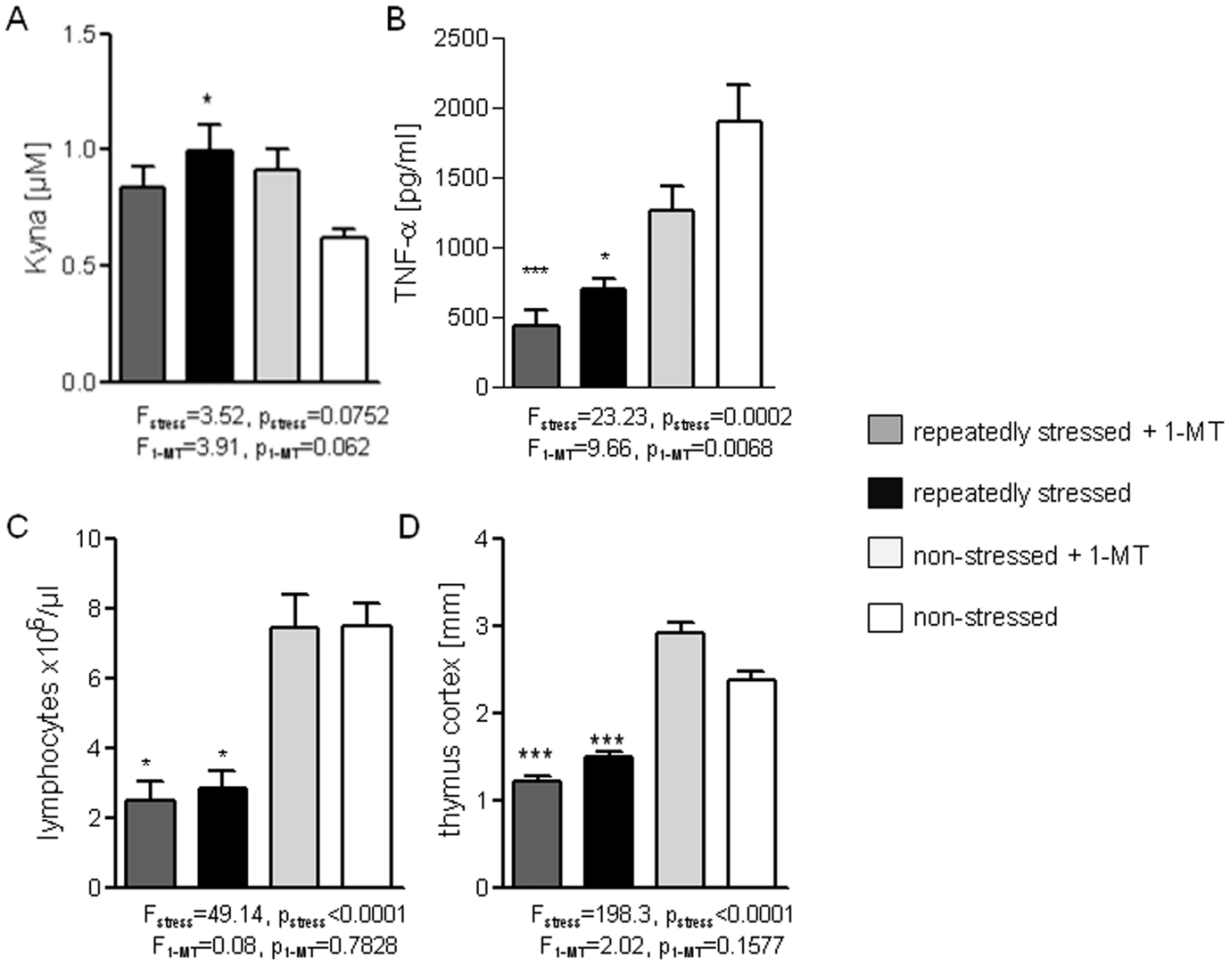
1-MT treatment: effects on Kyna levels, TNFα inducibility, lymphocytes and thymus cortex atrophy in repeatedly stressed mice. Plasma kynurenic acid concentrations (A), TNFα levels in the supernatant of splenocytes (B) after *ex vivo* stimulation with LPS (*from S. abortus equi*, 1-µg/ml), absolute lymphocyte counts in the blood (C) and thickness of the thymic cortex (D) of repeatedly stressed and non-stressed mice with or without 1-MT treatment; n = 8 mice/group, summary of two independent experiments; *p<.05, **p<.01, ***p<.001 compared with non-stressed, without 1-MT-treatment, ^#^p<.05; ^##^p<.01; ^###^p<.001 compared with repeatedly stressed mice by Kruskal-Wallis testing with *post-hoc* Dunn's Multiple comparison testing. Two-way ANOVA and *post-hoc* Bonferroni's Multiple comparison test were used for testing the overall influences of stress and treatment (ANOVA F- and p-values for the influence of stress and 1-MT are indicated in the graph).

Although the blockade of IDO1 activity significantly reduced the depletion of tryptophan, plasma levels of Kyna remained elevated after repeated stress (Fig. 4A). Separate analysis of Kyna levels in mice treated for 5-days with 1-MT vs. naïve mice revealed a significantly increase in plasma Kyna concentrations (1-MT: 0.916±0.088 µM vs. control: 0.616±0.038 µM, p = 0.045, n = 12 mice by Mann-Whitney test). This indicates that the 1-MT treatment causes Kyna accumulation by a yet unknown pathway. This went along with a reduced LPS-induced TNFα release of splenocytes (Fig. 4B), lymphocytopenia (Fig. 4C), and thymic atrophy (Fig. 4D) which were not affected by IDO1 blockade.

**Figure 5:**
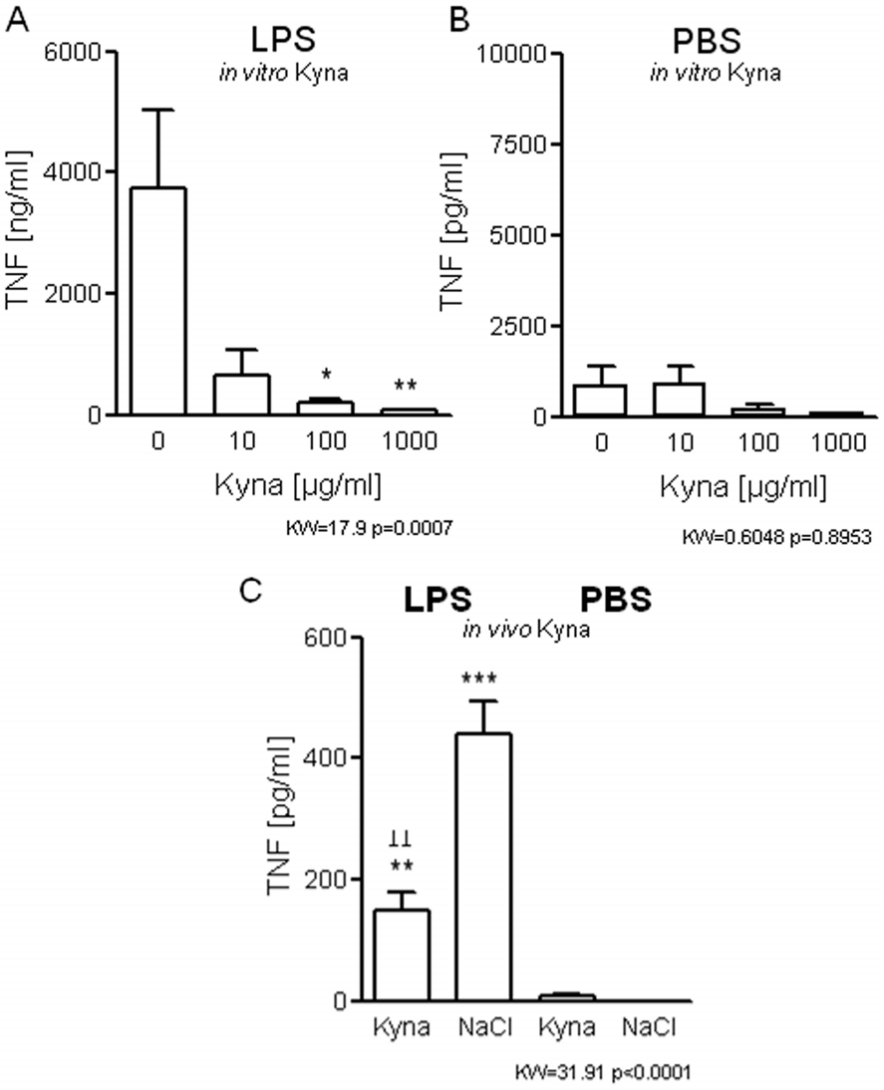
*In vivo* and i*n vitro* Kyna effects on TNFα inducibility, in naïve mice. Dose-dependent effects of Kyna on TNFα secretion into the supernatant (A) 16-h after stimulation with LPS (*E. coli* O55:B5, 500-ng/ml) or on spontaneous release in PBS-treated splenocyte cultures (B), and of LPS-stimulated splenocytes obtained from mice that were pre-treated with 250-ng Kyna/g BW or saline 6-h prior to *ex vivo* stimulation of splenocytes (C) n = 6 mice/group, summary of two independent experiments. *p<.05, **p<.01, ***p<.001 compared with NaCl control treatment, ^⊥⊥^<0.01 compared LPS control treatment by Kruskal-Wallis testing with *post-hoc* Dunn's Multiple comparison testing (KW- and p-values are indicated in the graph).

Reduced TNFα inducibility in mouse splenocytes is also found after *in vitro* Kyna treatment which is demonstrated by preventing the LPS-induced TNFα response (Fig. 5,A,B). In addition, we found that *in vivo* ip Kyna injection dampened the LPS-induced TNFα secretion in primary splenocyte cultures (Fig. 5C). Thus, high Kyna levels are capable to alter the TNFα responsivness *in vivo*.

### Enhanced Trp catabolism in patients during the peri-operative stress response

**Figure 6:**
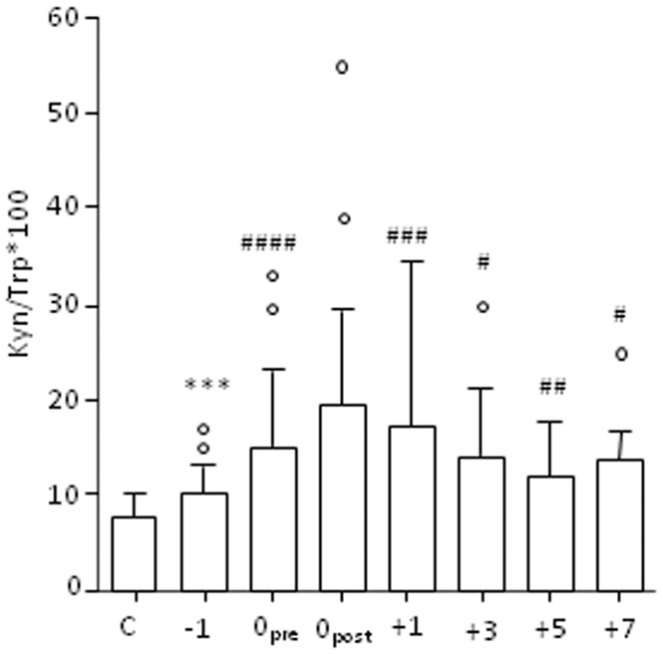
Tryptophan catabolism along the kynurenine pathway during the peri-operative stress response of patients undergoing elective abdominal surgery without complications. Preoperative, intraoperative and postoperative Kyn/Trp ratios in the plasma of 18 patients compared with Kyn/Trp ratios of 20 healthy volunteers ***p<.001 compared with healthy controls, ^#^p<.05, ^##^p<.01, ^###^p<.001, ^####^p<.0001 compared with values from pre-operative day -1.

To analyze whether psychological stress-induced activation of the kynurenine pathway is also relevant in humans, we conducted a pilot study monitoring the kinetics of Kyn/Trp ratio in the plasma of 18 patients prior to and after elective major abdominal surgery (esophagectomy, gastrectomy, pancreatic resection, colorectal resection) without postoperative complications. Although all patients received benzodiazepines to dampen the HPA axis reactivity prior to surgery, they all, independently of gender, presented an increased Kyn/Trp ratio already in the pre-operative period which remained increased up to 7-days after surgery (Fig. 6). Thus, activation of IDO1 activity was also measurable in patients who expected surgery and is even enhanced in the post-operative period which may contribute to postoperative immunosuppressive states.

## Discussion

### Trp catabolism: a role in stress-induced energy expenditure and immunosuppression in mice

In this study, we show for the first time that already an acute stress exposure causes a rapid up-regulation of IDO1 mRNA expression in different organs – most prominently in the brain. We found that sustained Trp catabolism reduces the anti-bacterial defense and induces behavioral alteration in repeatedly stressed BALB/c mice.

Although, we did not determine the cell population which induced IDO1 expression in our study, it was recently shown that IDO1 protein is detectable in a number of cells and tissues such as antigen-presenting cells of epididymis, placenta, spleen, thymus, lung and digestive tract [38] which remains to be investigated in the murine stress model.

Acute stress-induced up-regulation of IDO expression is likely linked to increased dissemination of commensal bacteria into mesenteric lymph nodes and liver which provokes a transiently increased proinflammatory cytokine bias in acutely stressed mice [37]. Other authors showed that pro-inflammatory cytokines such as TNFα and IFNγ induce IDO1 activity *in vitro* and *in vivo* [23], [28], [39]. In line with this, both TNFα and IFNγ blockade prevented the up-regulation of IDO1 expression in acutely stressed mice. We hypothesize that a TNFα-dependent IFNγ-inducing pathway is activated by stress due to increased bacterial load of extra-intestinal sites [37].

It was recently shown that TNFα up-regulates a whole set of enzymes including IDO1 supplying the nicotinamide adenine dinucleotide (NAD^+^) synthesis pathway (see Figure 1; [40]). We suggest that kynurenines which are generated in stressed mice are rapidly used for NAD^+^ synthesis because of a high energy demand in stressful situations [29], [40].

Moreover, our study revealed that prolonged activation of the Trp catabolic pathway during repeated stress alters the capacity to cope with infection in mice. Surprisingly, GCs which primarily activate the TDO-driven Trp catabolism [22], [27] don't have the leading role for Trp depletion or for the loss of the antibacterial defense in our model of repeated stress. In contrast, GCs seem to be essential for the early antibacterial response [41], [42] and, thus, blockade of GC receptor signaling was detrimental after experimental *E. coli* infection.

Two isoforms of IDO are described which both can be blocked by 1-Methyl-L-tryptophan [43] but there is evidence that only activation of the IDO1 isotype, not IDO2, alters immune function [43]. For the first time, our data show that L-1-MT treatment attenuated Trp depletion, restores the antibacterial defense and simultaneously reduces depression-like alterations in repeatedly stressed mice. It would be interesting to see whether IDO blockade could also prevent the spontaneous spreading of commensal bacteria into the body as shown in our previous studies [13].

The generation of pro-apoptotic kynurenines which preferentially affect T helper (Th)-1 and Th17 cells induce a Th2 and T regulatory (Treg)-driven anti-inflammatory cytokine bias [21], [22], [28] which is reflected in increased IL-10 levels after repeated stress. 1-MT treatment normalized the IL-10 release of LPS-stimulated splenocytes of stressed mice which was paralleled with an improved defense of experimental *E. coli* infection. We hypothesize that primarily increased IL-10 levels account for the reduced anti-bacterial defense after prolonged stressful experience than a reduced inducibility of TNFα which was not affected by 1-MT treatment in our study. The impact of IDO1 activation in repeated stress-induced loss of the anti-bacterial defense is underlined by the yet preliminary findings that stressed IDO1^−/−^mice were capable to cope with an *E.coli* infection comparable to non-stressed, wild-type mice (see Suppl. Fig. 4).

Supportively, IDO-induced formation of kynurenines in mice during mycobacterial infection alters the differentiation of Th1 and Th17 lymphocytes which increases the bacterial dissemination by reducing the availability of IFNγ which is essential of the antibacterial defense [44]. In addition, a positive correlation of IDO1 activity and plasma LPS levels in HIV-infected humans was linked to a decreased Th17/Treg ratio which is linked to disease progression [45]. Our results suggest, that prolonged stressful experiences may aggravate the risk of suffer from infections in different disease states via IDO1 activation.

To our surprise, 1-MT treatment did not prevent that Kyna levels increased in repeatedly stress mice and it also did not reconstitute the inducibility of TNFα in splenocytes. It was recently demonstrated that Kyna inhibits LPS-induced TNFα secretion in human peripheral blood mononuclear cells via gpr35 activation [46]. Thus, we hypothesize that peripherally increased Kyna concentration is a potent inhibitor of TNFα production *in vivo* in mice. Since Kyna can be produced by commensal bacteria and is found in pancreatic and bile juices [47], [48] formation of the molecule does not essentially dependent on IDO1 activity. Low-grade but significantly increased bacterial dissemination after intestinal barrier dysfunction [13], [37] may explain increased Kyna levels after repeated stress which may not be prevented by 1-MT treatment but is not investigated, yet.

Finally, in a preliminary study with a small sample size and mostly male patients, we demonstrate that patients undergoing surgery who suffer from increased anxiety, helplessness and social isolation show IDO1 activation [49], [50]. In the current study we didn't include measurements of the influence of physical stress due to the underlying disease which needs to be addressed in further studies but demonstrated that this patient group showed an activation of Trp catabolic pathways already in the pre-operative period which might contribute to the well-known increased infection risk and metabolic disturbances after major abdominal surgery [51]. In trauma patients, in fact, the Kyn/Trp ratio has recently been shown to be of predictive value for ongoing sepsis [52] and is under evaluation as target for preventing immunosuppression.

### Trp catabolism: a role in stress-induced behavioral alterations

Depletion of Trp, the precursor molecule of serotonin, promotes mood alterations in healthy individuals [29]–[31] and depression-like behavior in stressed animals [29]. In line with this, 1-MT treatment prevented both serotonin depletion and behavioral alterations. Increased intake of tryptophan leads to an acute increase of cerebral 5-HT and/or 5-HIAA in various species [29] and a rapid depletion of tryptophan which results in impaired cerebral 5-HT formation in mammals and humans [29], [53]. In line with this, 1-MT treatment increased the 5-HT/5-HIAA ratio in the brain of naïve mice and prevented that systemic Trp levels dropped-down in repeatedly stressed mice which was associated with reduced behavioral alterations.

In our study, we showed for the first time that activation of the Trp catabolism in BALB/c mice caused a peripheral accumulation of Kyna which is a (N-methyl-D-aspartat) NMDA receptor antagonist and is known to contribute to the development of disorders such as depression and schizophrenia [34]. Both, peripheral blood and central spinal fluid (CSF) concentration of kynurenine seem to correlate with the levels of Quin and Kyna in the CSF predicting depressive symptoms in patients after IFNα therapy [54]. This emphasizes the clinical relevance of our data giving supplementary evidence for a role of stress in the pathogenesis of mental and mood disorders.

### Conclusion

This study highlights a new stress-inducible pathway which adds up to the neuroendocrine circuits known to suppress immune functions: (1) acute stress-induced low-grade inflammation induces transient IDO1-mediated tryptophan-catabolism and (2) prolonged Trp shortage due to expanded stressful episodes as well as the formation of kynurenines are immunosuppressive and simultaneously may induce mood alterations.

## Materials and Methods

### Animals

Female BALB/c and IDO1^−/−^ mice (BALB/c background) derived from our own breeding facility (University of Greifswald, Germany). The female mice were kept in groups (6–9 mice/cage) under controlled conditions with 12/12 light cycle and food and water available *ad libitum*. Animal experiments were approved by the Animal Protection Committee of Mecklenburg-Vorpommern, Germany.

### Stress models

The repeated psychological stress model was described recently [7], [8], [13]. Mice at the age of 10–14-wks were exposed to combined acoustic and restraint stress for 4.5-days. Acute psychological stress was defined as a single 2-h-stress session in the morning to prevent influences of circadian rhythm on results. All successive analyses in mice with our without treaments were performed starting at 10 AM after the last stress exposure.

### Intervention studies

1-Methyl-L-tryptophan was administered in the drinking water (1-MT, 2-mg/ml) starting one day prior to the first stress session. Polyclonal TNFα-antiserum from sheep, IFNγ antiserum from rabbit (both kindly provided by G. Tiegs, University of Erlangen), or for vehicle normal sheep or rabbit serum (Roth, Karlsruhe, Germany) were administered ip at 200-µl volume. Sera/antisera were injected immediately prior to acute stress. Kynurenine (Kyn) and kynurenic acid (Kyna) (both from Sigma) were diluted in NaCl by ultrasound sonication, adjusted to pH 7.4. Kyn was injected ip at 100 µg/g BW and 30-min, 1-h and 2-h after administration plasma Trp catabolite levels were analyzed. Kyna was injected ip at 250 ng/g BW and 6-h later TNFα-inducibility of splenocytes was measured. The GC receptor blocker RU486 (from Sigma) was administered subcutaneously (sc) at 50 µg/g BW (diluted in cylodextrin, from Sigma) in 50 µl volume at 6 PM daily starting one day before the first stress session continuing during repeated stress exposure.

### Stress Severity Score (SSS)

The individual reaction of mice to each stress-exposure was monitored by an observational SSS as described in detail earlier [8]. After each stress-session the amount of released urine and feces in the tube was documented and assigned as a value of 0–6 points: no urination (0 points), wet area around urethra (1), wet parts of the ventral fur (2), wet ventral fur (3), additionally wet dorsal parts (4), wet body (5), completely wet animal (6); no feces (0), 1–5 pellets (1), 6–10 pellets (2) etc. up to more than 25 pellets (6). If diarrhea was detected, the value of defecation was doubled. After placing the animal back into its home cage the condition of fur was recorded (0–6 points): normal fur (0), fur of the head and the neck ruffled (1), dorsal fur partially ruffled (2), dorsal fur completely ruffled (3), additionally parts of the ventral fur ruffled (4), dorsal and ventral fur ruffled (6), whole animal's fur strongly ruffled (6). Activity scores (locomotor activity, explorative activity, huddling, 0–6 points), and muscle tone (0–3 points) were also estimated: normal activity (0 points), slightly reduced exploration (1), reduced exploration with short intervals without activity (2), longer intervals without activity (3), strongly reduced activity and huddling (4), huddling and lethargy but reaction to environmental stimuli (5), no reaction to environmental stimuli (6); normal muscle tone (0 points), slightly reduced muscle tone with temporary drop down of the tail (1), reduced muscle tone with longer intervals of trailing the tail (2), strongly reduced muscle tone with a constant trailing of the tail during the first 5 min after bringing the mice back into the home cage (3). The SSS for each mouse finally consists of points rated by the investigator during nine stress cycles ranging from 0 to 297 points of maximum performance.

### Harvesting of blood and organ samples

For anesthesia, 75-µg/g BW Ketamin Curamed® (CuraMED Pharma GmbH, Karlsruhe, Germany) and 16-µg/g BW Rompun® (Bayer AG, Leverkusen, Germany) were diluted in pyrogen-free 0.9% sodium chloride (Braun, Melsungen, Germany) and injected intraperitoneally prior to harvesting of blood by retroorbital puncture. K_2_E-EDTA-plasma samples were stored at -20°C until use. Spleens were aseptically removed and put into sterile cold VLE RPMI 1640 medium (Biochrom KG Berlin, Germany) and single cell suspensions for inducing cytokine production were prepared within one hour after organ harvesting. For histological analysis thymus was isolated, immediately frozen in Tissue-Tek® (O.C.T.TM compound, Sakura, Netherlands) in liquid nitrogen and then stored at −80°C until use.

For analysis of gene expression whole brain, lung, liver and the first 4–5 Peyer's patches (PPs) proximal of the ileocecal junction (dissected from the ileal tissue) were removed and immediately frozen in liquid nitrogen.

### Lymphocyte counts and e*x vivo* inducibility of cytokines

Absolute lymphocyte numbers in whole blood were analyzed by a hemocounter (Sysmex K-4500, SYSMEX GmbH, Germany). Splenocytes were adjusted to 1×10^7^ cells/ml in culture medium (VLE RPMI 1640 from Biochrom KG, Berlin, Germany; with 200 mM N-acetyl-L-alanyl-L-glutamine from Biochrom KG, Berlin, Germany; 100 mM Na-pyruvate from GIBCO BRL, Paisley, Scotland; 2% D(+)-glucose from Sigma, Steinheim, Germany, 10% FCS from Biochrom KG, Berlin, Germany). 500 µl of cell suspensions were transferred into sterile covered 5 ml-tubes (Greiner, Frickenhausen, Germany). For measuring cytokine inducibility lipopolysaccharide was added to the cultures with a final concentration of 1 µg/ml LPS from *S. abortus equi* or 500 ng/ml LPS form *E. coli* O55:B5. For studying *in vitro* effects of Kyna, the catabolite was added to get 0, 10, 100, 100 µM solutions (all chemicals from Sigma). Cells were incubated for 42-h at 37°C and 5% CO_2_. Supernatants were analyzed by Inflammation-Cytometric Beads Array following the manufacturer's protocol (CBATM, BD Pharmingen).

### Histological analysis

Frozen sections (6-µm thin) from thymus were stained by Hemalaun + Eosin (H/E) using conventional protocols and thymic cortex diameter (10 measurements/section) was analyzed by Metamorph Imaging software 4.5.

### Infection with *E. coli*

Mice were infected by intraperitoneal injection (ip) with 3×10^5^ cfu of *E. coli* ATCC 25922. 24-h later bacterial load of liver and blood was determined by directly plating homogenates or blood on Columbia agar plates (BD, Heidelberg, Germany).

### IDO1 mRNA expression

Total RNA was isolated using Trizol Reagent (Invitrogen, Ontario, CA). All tissue samples were separately homogenized on ice in sterile tubes containing 1 ml of ice-cold Trizol Reagent per 50–100 mg using a homogenizer at 24,000 rpm for 3-min. Homogenized samples were incubated for 15-min at RT. Bromo-chloro-propan (0.2 ml, Sigma, Germany) was added to 1 ml Trizol reagent and vigorously shaken for 30-s, incubated for 10-min at RT and centrifuged at 9,500 rpm for 15-min at 4°C. The aqueous phase was transferred to a fresh tube and RNA was precipitated by adding 0.5 ml isopropanol (Sigma, Germany). Samples were incubated for 15-min at RT and centrifuged at 9,500 rpm for 10-min at 4°C. RNA pellet was briefly dried and dissolved in RNase-free water and stored at −80°C until use.

Reverse transcription of mRNA was performed with the mouse MGB IDO1 primer mixture (Gene Expression Assays: Mm00492586_m1) and mouse GAPDH primers (Applied Biosystems, Foster City, CA). Real-time PCR was carried out in an ABI PRISM 7000 SDS (50°C, 2-min and 95°C, 10-min, 40-cycles 95°C,15-s and 60°C 1-min; Applied Biosystems). IDO1 mRNA was quantified by Ct method normalized to GAPDH using ABI Prism 7000 software. (GAPDH expression in control mice and acutely stressed animals was comparable, e.g. in brain IDO1-signal detectable at cycle: 17.33±1.46 in non-stressed mice, 16.83±1.73 at 2-h and 16.38±0.58 6-h after acute stress). Expression of IDO1mRNA was measured in whole brain, lung and liver homogenates and in homogenates of 4–5 isolated Peyer's patches (PPs).

### Quantification of plasma indoleamines

Concentrations of tryptophan (Trp), kynurenine (Kyn), kynurenic acid (Kyna), quinolinic acid (Quin), and serotonin in plasma samples were analyzed by tandem mass spectrometry. 100 µl samples of plasma were analyzed after adding 50 µl sulfosalicylic acid (Sigma, 10-g in 90-mlmethanol), 40 µl water and 10 µl internal standard (d5-Trp, and d5-Kyna, Euriso-top, Paris, France). Samples stored at 4°C for 30-min and centrifuged (20,000-g, 10-min). Supernatant was measured. A Wallac MS2 tandem mass spectrometer (Perkin Elmer, Rodgau, Germany) equipped with an electrospray ion source was used to record Trp catabolites and standards. Ions were detected in a positive ion mode using multiple reaction monitoring. Calibration curves were fitted by linear least-square regression and correlated to the concentration of d5-Trp and d5-Kyna as internal standards.

### Brain serotonin turnover

Concentrations of serotonin and 5-HIAA in brain were determined by HPLC with electrochemical detection. Samples of half mouse brains were homogenised in 4 ml of 0.2 M perchloric acid followed by centrifugation at 45,000-g at 4°C. Pooled supernatants of two repeated extractions were again centrifuged at 45,000-g and aliquots of 20 µl were injected into the HPLC system, equipped with a 125×4 mm column packed with Prontosil C18 AQ (Bischoff Analysentechnik, Leonberg, Germany). The mobile phase consisted of 58 mM sodium hydrogen phosphate buffer containing 1.2 mM octanesulfonic acid, 0.3 mM EDTA, 0.2 mM potassium chloride and 6% methanol at pH 3.5. Electrochemical dectection was achieved by an ISAAC cell with a glassy carbon working electrode set at a potential of 600 mV (Axel Semrau, Sprockhövel, Germany). As an index of serotonin turnover, the ratio between 5-HIAA and serotonin concentrations was calculated.

### Human peri-operative stress response

18 patients (15 male, 3 female, 37–89 yrs, Caucasian ethnicity) who underwent elective major surgery of the gastrointestinal tract (esophagectomy, gastrectomy, pancreatic resection, colorectal resection mostly related to cancer) at the University hospital of Greifswald were enrolled in a prospective observational study to analyze indoleamines. Inclusion criteria comprised given informed consent for participation and age >18 years. The exclusion criteria were pregnancy, immunosuppression from cytostatic therapy, high-dosage steroid therapy, or moribund state. Patients or their authorized representatives gave written informed consent. The study was approved by the local Institutional Review Board. At baseline, demographic variables were collected. To dampen their preoperative stress response, they received benzodiazepines as pre-anaesthetic medication (Midazolam 3.75 to 7.5 mg) one day and 2-h before surgery. They were anesthetized with Remifentanyl, Propofol i.v. (duration of operation: 185–497 min). For postoperative pain relief, all patients received anaesthetics (0.2% Ropivacain, 0.5 µg/ml Sufentanyl) through a peridural catheter. Blood sample were collected in the morning of the preoperative day (-1), preoperatively (0_pre_), postoperatively (0_post_) and in the morning of postoperative days +1, +3, +5 and +7. Blood was collected in K_2_E-EDTA vacutainers (BD VacutainerTM, Plymouth, UK). Plasma samples were stored at −20°C until use. Plasma indoleamine concentrations were measured described above. Data were compared to those of 20 age-matched healthy controls (15 male, 5 female, 27–85 yrs, Caucasian ethnicity).

### Statistical analysis

Statistical analysis was carried out with GraphPad Prism 5 (GraphPad Software Inc., San Diego, CA, USA). Differences between two groups were analyzed by the Mann-Whitney U-test and of more than two groups without additional treatment by Kruskal-Wallis test with *post hoc* Dunn's Multiple Comparison test which are non-parametric tests not assuming Gaussian distribution. Comparison of data from stressed and non-stressed mice in studies with additional treatment was performed by two-way ANOVA and *post hoc* Bonferroni's Multiple Comparison test. Kruskal Wallis statistics (KW) or Bonferroni F-values (F) and p-values are indicated in the graphs. The data are depicted as mean ± SEM; p<0.05 was considered statistically significant. If not differentially indicated, data are representative of at least two independent experiments.

## Supporting Information

Figure S1 Kyn-degradation pathway a in mice. Healthy female mice were ip injected with Kyn at concentration of 100 µg/g BW. At 30-min, 1-h and 2-h after injection mice were sacrified and plasma levels of kynurenine (Kyn), kynurenic acid (Kyna) and quinolinic acid (Quin) were quantified. Data from 3 mice/time of a pilot study were shown. *p<.05 compared with non-stressed mice by Wilcoxon-test.
(0.03 MB TIF)

Figure S2 Effects of pharmacological glucocorticoid receptor blockade by RU-486 on plasma Trp levels and antibacterial defense in repeatedly stressed BALB/c mice. Plasma Trp concentrations in chronically stressed and non-stressed animals which were treated with RU486 or vehicle cyclodextrine (A); and bacterial burden in the spleen (B) blood 24-h (C) after experimental ip infection with 3×10∧5 cfu of E.coli ATCC25922 in repeatedly stressed (black bars) or non-stressed mice (white bars) that were daily treated with RU486 (50 µg/g BW/d, sc) or the vehicle. n = 6 mice/group. *p<.05, **p<.01, ***p<.001 compared with non-stressed, without 1-MT-treatment, #p<.05; ##p<.01; ###p<.001 compared with repeatedly stressed mice by Kruskal-Wallis testing with post-hoc Dunn's Multiple comparison testing. Two-way ANOVA and post-hoc Bonferroni's Multiple comparison test were used for testing the overall influences of stress and treatment (ANOVA F- and p-values for the influence of stress and 1-MT are indicated in the graph).
(0.07 MB TIF)

Figure S3 Proof of pharmacological 1-MT treatment in mice. Serotonin turnover in the brain of healthy 1-MT treated and non-treated mice shown as ratio between 5-hydroxyindole-3-acetic acid (5-HIAA) and serotonin; n = 5 mice/group, representative example of two independent experiments. *p<.05 compared with non-treated mice by Mann-Whitney test.
(0.02 MB TIF)

Figure S4 Bacterial dissemination in repeatedly stressed and non-stressed IDO knock-out mice. Bacterial burden in liver (A) and blood (B) 24-h after ip infection with E. coli ATCC 25922 in mice with or without 1-MT treatment after repeated stress and of non-stressed mice (injection was performed immediately after the ninth stress exposure); n = 6 mice/group result from one preliminary experiment; number of mice showing detectable bacterial load/mice in group is indicated in the graphs; *p<.05 compared with non-stressed, without 1-MT-treatment, #p<.05 compared with repeatedly stressed mice by Kruskal-Wallis testing with post-hoc Dunn's Multiple comparison testing.
(0.04 MB TIF)
