# Supplementary figures and images for: Psychological Stress-Induced, IDO1-Dependent Tryptophan Catabolism: Implications on Immunosuppression in Mice and Humans
Source: PLoS One. 2010 Jul 28;5(7):e11825. doi: 10.1371/journal.pone.0011825 (PMC2911374; doi:10.1371/journal.pone.0011825)

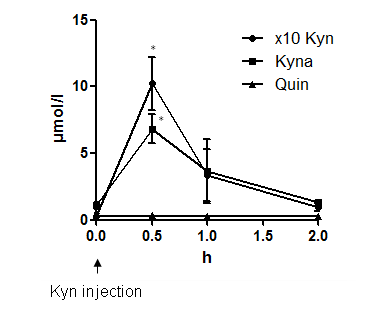

Supplement: Figure S1 — Kyn-degradation pathway a in mice. Healthy female mice were ip injected with Kyn at concentration of 100 µg/g BW. At 30-min, 1-h and 2-h after injection mice were sacrified and plasma levels of kynurenine (Kyn), kynurenic acid (Kyna) and quinolinic acid (Quin) were quantified. Data from 3 mice/time of a pilot study were shown. *p<.05 compared with non-stressed mice by Wilcoxon-test. (0.03 MB TIF) [file pone.0011825.s001.tif]

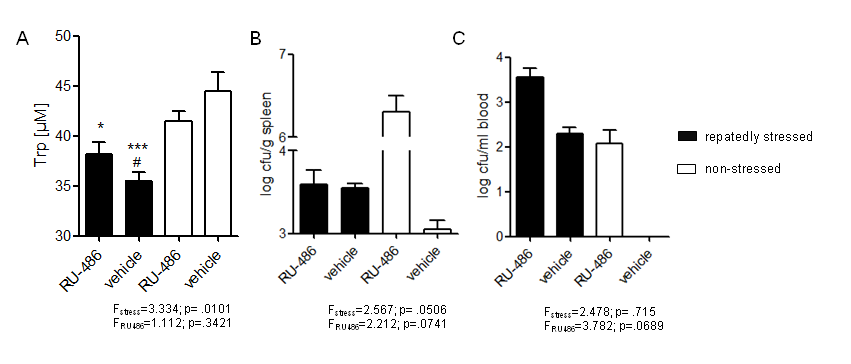

Supplement: Figure S2 — Effects of pharmacological glucocorticoid receptor blockade by RU-486 on plasma Trp levels and antibacterial defense in repeatedly stressed BALB/c mice. Plasma Trp concentrations in chronically stressed and non-stressed animals which were treated with RU486 or vehicle cyclodextrine (A); and bacterial burden in the spleen (B) blood 24-h (C) after experimental ip infection with 3×10∧5 cfu of E.coli ATCC25922 in repeatedly stressed (black bars) or non-stressed mice (white bars) that were daily treated with RU486 (50 µg/g BW/d, sc) or the vehicle. n = 6 mice/group. *p<.05, **p<.01, ***p<.001 compared with non-stressed, without 1-MT-treatment, #p<.05; ##p<.01; ###p<.001 compared with repeatedly stressed mice by Kruskal-Wallis testing with post-hoc Dunn's Multiple comparison testing. Two-way ANOVA and post-hoc Bonferroni's Multiple comparison test were used for testing the overall influences of stress and treatment (ANOVA F- and p-values for the influence of stress and 1-MT are indicated in the graph). (0.07 MB TIF) [file pone.0011825.s002.tif]

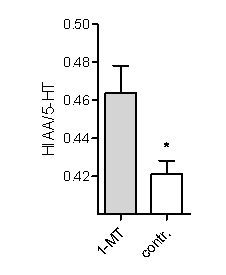

Supplement: Figure S3 — Proof of pharmacological 1-MT treatment in mice. Serotonin turnover in the brain of healthy 1-MT treated and non-treated mice shown as ratio between 5-hydroxyindole-3-acetic acid (5-HIAA) and serotonin; n = 5 mice/group, representative example of two independent experiments. *p<.05 compared with non-treated mice by Mann-Whitney test. (0.02 MB TIF) [file pone.0011825.s003.tif]

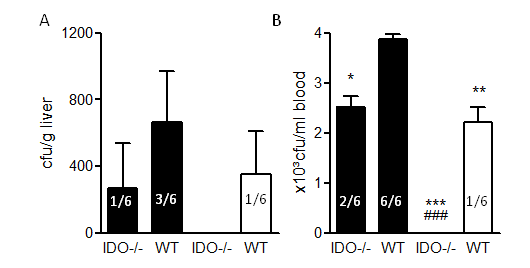

Supplement: Figure S4 — Bacterial dissemination in repeatedly stressed and non-stressed IDO knock-out mice. Bacterial burden in liver (A) and blood (B) 24-h after ip infection with E. coli ATCC 25922 in mice with or without 1-MT treatment after repeated stress and of non-stressed mice (injection was performed immediately after the ninth stress exposure); n = 6 mice/group result from one preliminary experiment; number of mice showing detectable bacterial load/mice in group is indicated in the graphs; *p<.05 compared with non-stressed, without 1-MT-treatment, #p<.05 compared with repeatedly stressed mice by Kruskal-Wallis testing with post-hoc Dunn's Multiple comparison testing. (0.04 MB TIF) [file pone.0011825.s004.tif]
